# Extremity Snakebite in Routine Clinical Practice: Clinical, Laboratory, and Treatment-Timing Characteristics of Patients Managed with and Without Fasciotomy

**DOI:** 10.3390/jcm15187260

**Published:** 2026-09-18

**Authors:** Güneş Sarıkaya, Mücahit Çelik, Fatih Işık, Özgün Karakuş

**Affiliations:** 1 Orthopedics and Traumatology Clinic, Balıkesir Atatürk City Hospital, Balıkesir 10100, Türkiye; 2 Orthopedics and Traumatology Clinic, Balıkesir Atatürk City Hospital, University of Health Sciences Türkiye, Balıkesir 10100, Türkiye

**Keywords:** snake bites, fasciotomy, antivenins, clinical decision-making, compartment syndromes

## Abstract

**Introduction**: Severe local envenomation following extremity snakebite may mimic acute compartment syndrome and complicate surgical assessment. We aimed to describe the clinical, laboratory, and treatment-timing characteristics of patients managed with and without fasciotomy. **Methods**: This single-center retrospective study included patients with extremity snakebite treated between 2023 and 2025. A six-item clinical profile score was constructed retrospectively to summarize documented local findings: pain at rest, pain with passive motion, delayed capillary refill, marked swelling or tenseness, paresthesia, and motor deficit (1 point for documented presence, 0 for documented absence; range, 0–6). Clinical, laboratory, and treatment-timing characteristics were compared between patients managed with and without fasciotomy; analyses were exploratory and interpreted descriptively. **Results**: The overall cohort included 50 patients, six of whom underwent fasciotomy. Antivenom administration was documented in 25 patients, absent in 14, and unknown in 11. The complete-case subset comprised 35 patients, including 21 antivenom-treated patients. Within this subset, patients undergoing fasciotomy had higher clinical profile scores (median, 6 vs. 1) and longer bite-to-presentation intervals (median, 4.5 vs. 2 h; both *p* < 0.001). No significant between-group difference was detected in emergency department arrival-to-antivenom intervals (*p* = 0.129). Platelet counts were lower and activated partial thromboplastin time was longer in the fasciotomy group. Median bite-to-fasciotomy and antivenom-to-fasciotomy intervals were 8 and 2 h, respectively. **Conclusions**: Patients selected for fasciotomy had more pronounced documented local findings and later presentation. These exploratory associations do not establish surgical necessity or pressure-confirmed acute compartment syndrome; the retrospectively constructed clinical profile score remains descriptive, without established diagnostic validity.

## 1. Introduction

Snakebite envenoming is a neglected public health problem that causes substantial morbidity and mortality worldwide [1]. Snakebites most commonly involve the extremities, where local envenomation may present with pain, progressive swelling, tenseness, tissue injury, and neurovascular findings [2]. These findings may influence emergency department monitoring, the need for antivenom, and the decision to obtain surgical evaluation.

In the limbs, the deep fascia forms a connective tissue sheath overlying muscles and gives rise to intermuscular septa that contribute to the organization of muscular compartments. Histologically, it comprises two to three layers of collagen fiber bundles with differing orientations, separated by thin layers of loose connective tissue that permit sliding between layers [3]. Snake venom can cause direct tissue and microvascular injury, increased vascular permeability, and secondary inflammation, resulting in local edema and pain [2,4]. These effects may produce marked swelling, tenseness, and sensory disturbances that mimic acute compartment syndrome without necessarily indicating pressure-related impairment of tissue perfusion [2,4]. However, recent literature emphasizes that true venom-induced compartment syndrome is uncommon and that the use of intracompartmental pressure measurement and fasciotomy varies across studies and centers [5,6]. In a US snakebite registry study, compartment syndrome was suspected in 22 patients, representing approximately 1% of all reported cases; nine of these 22 patients underwent fasciotomy [6]. This uncertainty highlights the need to better characterize the clinical profile of patients who undergo fasciotomy, particularly in practice settings where objective pressure measurement is not routinely used.

To address this gap, we aimed to describe the clinical, laboratory, and treatment-timing characteristics of patients with extremity snakebite managed with and without fasciotomy in routine practice.

## 2. Materials and Methods

### 2.1. Study Design, Setting, and Ethical Approval

This single-center retrospective observational study was conducted by reviewing the medical records of patients who presented with extremity involvement after snakebite at a tertiary care hospital between 2023 and 2025. The objective was to describe the clinical, laboratory, and treatment-timing characteristics of patients with extremity snakebite managed with and without fasciotomy in routine clinical practice. Between-group comparisons were considered exploratory and descriptive because of the small number of fasciotomy cases.

The study was approved by the relevant Institutional Ethics Committee (Decision No. 2025/08/86). Because of the retrospective design, the requirement for informed consent was waived.

### 2.2. Patient Identification and Eligibility Criteria

Patients were identified by screening the hospital information management system for records coded with ICD-10 T63.0, corresponding to the toxic effect of snake venom. Available emergency department, inpatient, operative, and outpatient clinical records were reviewed to confirm snakebite-related extremity involvement and extract study variables. Patients presenting during the study period with extremity snakebite were eligible for the overall cohort. Non-extremity bites and presentations ultimately attributed to non-snake traumatic or dermatologic conditions were excluded. Missing clinical or treatment-timing data did not preclude inclusion in the overall cohort. A complete-case subset was defined by the availability of the predefined local clinical findings, bite-to-presentation time, and reliable antivenom timing and total dose data where applicable. Complete-case status referred to the availability of these analysis variables, not to the completeness or duration of postoperative follow-up. Available clinical follow-up records were reviewed without requiring a standardized follow-up schedule or minimum follow-up duration.

### 2.3. Case Selection Flow

**Figure 1:**
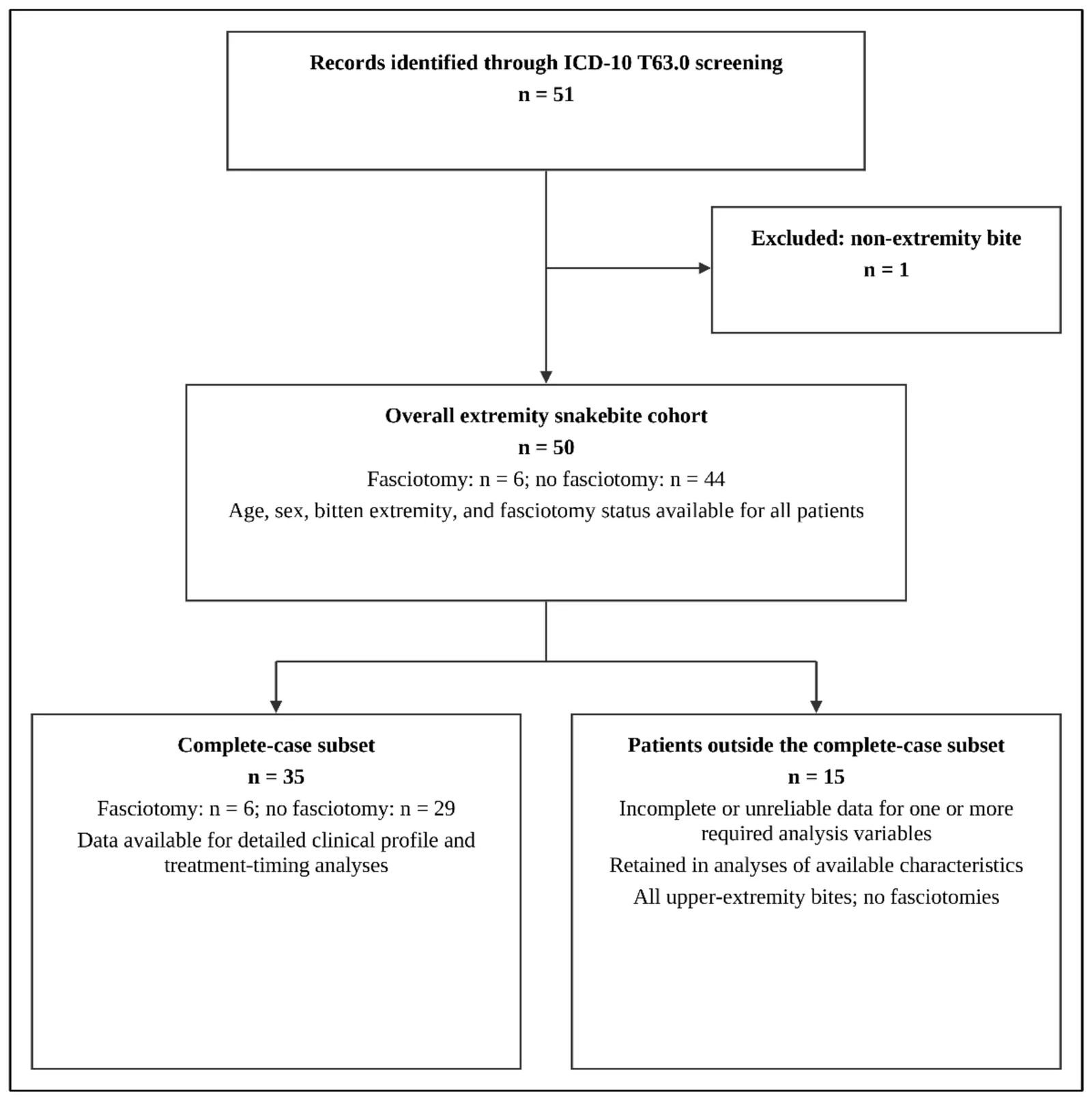
Flow diagram of the overall cohort and complete-case subset.

During the study period, 51 records coded as ICD-10 T63.0 were identified. One patient was excluded because the bite did not involve an extremity, leaving an overall cohort of 50 patients, six of whom underwent fasciotomy. Age, sex, bitten extremity, and fasciotomy status were available for all 50 patients. Thirty-five patients constituted the complete-case subset for detailed clinical profile and treatment-timing analyses. The remaining 15 patients had incomplete or unreliable data for one or more required variables but were retained in analyses of available characteristics. All 15 had upper-extremity bites, and none underwent fasciotomy. Antivenom administration was documented in four of these patients, although dose and timing data were unavailable; administration status could not be determined for the other 11. Antivenom administration status was therefore known for 39 patients, whereas dose and timing analyses included the 21 antivenom-treated patients in the complete-case subset. The patient selection process is summarized in Figure 1.

Antivenom administration status was known in 39 patients and unknown in 11. Dose and timing analyses included 21 treated patients in the complete-case subset; four additional patients had documented antivenom administration but lacked reliable dose and timing data.

### 2.4. Management Approach, Snake Species, and Antivenom

Clinical management was performed by emergency physicians using the clinical severity-based treatment framework described by the Turkish Emergency Medicine Association Toxicology Working Group [7]. For patients in whom antivenom administration was considered, case-specific recommendations were obtained from the National Poison Consultation Center. Treatment decisions were guided by the clinical and laboratory severity and progression of envenomation rather than by confirmed snake species.

The antivenom used in this cohort was the HSGM Polyvalent Snake Antivenom, owned by the General Directorate of Public Health of the Ministry of Health of Türkiye. The product was manufactured at Albila Serum Biological Products Co., Ltd. (Eskişehir, Türkiye), and vial filling was performed by Nobel Pharmaceuticals (Istanbul, Türkiye). Each vial contained 10 mL of injectable solution. The antivenom is produced using venoms from *Vipera ammodytes montandoni*, *Macrovipera lebetina obtusa*, and *Montivipera xanthina* [8]. The referenced framework recommends withholding antivenom when there are no local or systemic manifestations (grade 0) or when swelling is mild, without systemic manifestations or laboratory abnormalities (grade 1), unless edema progresses, in which case 1 vial may be administered. It recommends 2–4 vials for moderate envenomation (grade 2), characterized by increasing swelling, pain, and ecchymosis with mild systemic manifestations and laboratory abnormalities, and 4–6 vials for severe envenomation (grade 3), characterized by progressive local injury and severe systemic manifestations, such as marked thrombocytopenia, coagulopathy, or renal failure [7]. It recommends dilution in normal saline and intravenous administration under close monitoring [7]. In routine practice, decisions to administer additional vials were made by emergency physicians in consultation with the National Poison Consultation Center, based on serial clinical and laboratory assessments and continued progression of local or systemic findings.

Reliable cumulative antivenom dose and timing data were available for the 21 antivenom-treated patients in the complete-case subset and were used in the corresponding analyses. However, a formal envenomation grade, the specific clinical rationale for each additional dose, and the patient-level route of administration were not consistently documented in the retrospective medical records. Therefore, patient-level grading, grade-specific dose concordance, and route-specific analyses could not be performed. No confirmed, probable, photograph-based, or patient-description-based snake identification was available for any patient in the complete-case cohort. Accordingly, all 35 cases were classified as completely unidentified, and species-based subgroup analysis was not performed.

### 2.5. Data Collection and Timing Variables

Demographic characteristics, bite-to-presentation time, anatomical site of the bite, documented local clinical findings, laboratory results, antivenom administration, bite-to-antivenom interval, ED arrival-to-antivenom interval, total antivenom dose, fasciotomy performance, time to fasciotomy, antivenom-to-fasciotomy interval, postoperative complications, and hospital length of stay were extracted from the electronic medical records. Bite-to-presentation time was defined as the interval between the patient-reported time of snakebite and arrival at the emergency department. Among patients who received antivenom, the bite-to-antivenom interval was defined as the interval between the patient-reported time of snakebite and initiation of antivenom infusion. The ED arrival-to-antivenom interval was calculated as the difference between the bite-to-antivenom interval and the bite-to-presentation time. All timing variables, including bite-to-presentation time, bite-to-antivenom interval, ED arrival-to-antivenom interval, time to fasciotomy, and antivenom-to-fasciotomy interval, were recorded and analyzed in hours. Total antivenom dose was defined as the cumulative number of antivenom vials administered during the hospital course. Among patients who underwent fasciotomy, time to fasciotomy was defined as the interval between the patient-reported time of snakebite and the fasciotomy procedure. The antivenom-to-fasciotomy interval was calculated among patients who received antivenom before fasciotomy. Data extraction was performed using predefined variables, and records were reviewed independently by two investigators; discrepancies were resolved by consensus with a third investigator.

For the six patients who underwent fasciotomy, additional patient-level information was extracted, when available, regarding the specific bite location, documented reason for proceeding to surgery, anatomical extent of fasciotomy, intraoperative findings including muscle viability, tissue necrosis and debridement, wound closure and skin grafting, postoperative neurovascular status, infection and other complications, additional surgery after grafting, amputation, range of motion, contracture, and functional recovery. Available inpatient and outpatient postoperative records were reviewed; however, there was no prespecified or standardized follow-up schedule, and the timing and duration of follow-up could not be reliably determined on a patient-by-patient basis. These patient-level variables were reported descriptively without additional statistical analysis.

A six-item clinical profile score was derived from emergency department records and orthopedic consultation notes to summarize local extremity findings documented during early emergency department assessment and/or orthopedic consultation before the final management decision. The six items were pain at rest, pain with passive motion, delayed capillary refill, marked swelling or tenseness, paresthesia, and motor deficit. These components were selected because they were regularly documented in the available clinical records, allowing for a consistent retrospective description of the local clinical profile.

Pain at rest and pain with passive motion were coded according to explicit documentation of pain at rest and pain elicited by passive movement, respectively. Delayed capillary refill was defined as a capillary refill time exceeding 2 s, based on the documented clinical assessment. Marked swelling or tenseness was coded as present only when the examination notes explicitly described swelling or tenseness as marked, severe, or clinically prominent. Paresthesia was based on documented patient-reported symptoms, whereas motor deficit was coded only when motor loss or weakness was explicitly recorded by the examining physician. The score was calculated as an unweighted count of documented positive findings, assigning 1 point for documented presence and 0 points for documented absence of each item (total range, 0–6). This approach does not imply equal clinical importance or diagnostic value across components. Missing documentation was not coded as absence. The score was constructed retrospectively for research purposes to summarize the documented local clinical profile; it was not part of the contemporaneous clinical assessment and was not used to guide treatment or fasciotomy decisions.

### 2.6. Outcome Definitions

The primary outcome was fasciotomy performance during the hospital course. Fasciotomy performance was defined as the documentation of fasciotomy in the operative record or clinical follow-up notes after orthopedic evaluation. Fasciotomy was analyzed as an observed management outcome.

Intracompartmental pressure measurements were not routinely performed or documented. Secondary outcomes included hospital length of stay and, among patients who underwent fasciotomy, documented postoperative complications, reoperation, amputation, and postoperative neurovascular status.

### 2.7. Laboratory Evaluation

Admission laboratory parameters selected for the focused analysis included hemoglobin, platelet count, international normalized ratio (INR), activated partial thromboplastin time (aPTT), creatine kinase (CK), and serum creatinine. The first available laboratory value obtained at admission or on the same calendar day was used. Hemoglobin, platelet count, INR, aPTT, CK, and creatinine were available for all 35 patients. Fibrinogen and D-dimer were available for only 7 (20.0%) and 12 (34.3%) patients, respectively, and were therefore not included in between-group comparisons. Separate prothrombin time and fibrin degradation product measurements were not consistently available and were not analyzed.

### 2.8. Statistical Analysis

Statistical analyses were performed using IBM SPSS Statistics for Windows, version 26.0 (IBM Corp., Armonk, NY, USA). Continuous variables were assessed for distributional characteristics and are presented as median and interquartile range. Categorical variables are presented as numbers and percentages. Patients were compared according to fasciotomy management using the available data for each analysis. Age, sex, and bitten extremity were analyzed in the overall cohort (n = 50). Antivenom administration was compared among patients with known administration status (n = 39); unknown status was not classified as non-administration. Clinical profile, laboratory, presentation-timing, and hospital length-of-stay analyses used the complete-case subset (n = 35). Antivenom dose and timing analyses were restricted to treated patients within this subset (n = 21). Continuous variables were compared using the Mann–Whitney U test, and categorical variables using two-sided Fisher’s exact tests. Exploratory correlations between the clinical profile score and selected continuous variables were assessed using Spearman’s rank correlation coefficient. Because only six patients underwent fasciotomy, all between-group comparisons were considered exploratory and were interpreted descriptively. No multivariable regression model was constructed, and no data-driven diagnostic or surgical decision threshold was derived from the clinical profile score. All tests were two-sided, and a *p*-value < 0.05 was used as the nominal significance threshold. Given the exploratory nature of the study, no adjustment was made for multiple comparisons. Accordingly, all reported *p*-values are unadjusted and should be interpreted cautiously.

## 3. Results

### 3.1. Study Population and Baseline Characteristics

The overall cohort comprised 50 patients with extremity snakebite, of whom six (12.0%) underwent fasciotomy. The median age was 47 years (interquartile range [IQR], 29.5–60), 20 patients (40.0%) were female, and 37 (74.0%) had upper-extremity bites. Antivenom administration was documented in 25 patients (50.0%), whereas 14 (28.0%) did not receive antivenom, and administration status could not be determined for 11 (22.0%).

Thirty-five patients formed the complete-case subset for detailed analyses, including all six fasciotomy patients and 29 patients managed without fasciotomy (Figure 1). Of these, 21 received antivenom and had reliable dose and timing data. Snake species could not be identified in any patient in this subset. Characteristics of the overall cohort and complete-case subset are summarized in Table 1.

### 3.2. Individual Documented Local Clinical Findings During Early Clinical Evaluation

Individual documented local clinical findings recorded during emergency department assessment and/or orthopedic consultation before the final management decision are shown in Table 2. Pain with passive motion was documented in all patients in both groups (6/6 vs. 29/29). In contrast, pain at rest (6/6 vs. 3/29), marked swelling or tenseness (6/6 vs. 3/29), paresthesia (6/6 vs. 2/29), motor deficit (6/6 vs. 0/29), and delayed capillary refill (5/6 vs. 0/29) were more frequently documented among patients who underwent fasciotomy.

### 3.3. Other Characteristics According to Fasciotomy Management

Comparisons of demographic, treatment-timing, laboratory, and hospital-course characteristics according to fasciotomy management are presented in Table 3. In the overall cohort, no statistically significant differences were detected in age, sex, or bitten extremity between management groups. Antivenom administration also did not differ significantly among patients with known administration status. Within the complete-case subset, the following treatment-timing, laboratory, and hospital-course findings were observed. Patients who underwent fasciotomy presented later after the bite than those managed without fasciotomy (4.5 h [IQR, 4–5] vs. 2 h [IQR, 2–2], *p* < 0.001). Among patients who received antivenom, the bite-to-antivenom interval was longer in the fasciotomy group (4.5 h [IQR, 4–5] vs. 2 h [IQR, 2–3], *p* < 0.001). However, the ED arrival-to-antivenom interval was not significantly different between the groups (0 h [IQR, 0–0] vs. 0 h [IQR, 0–1], *p* = 0.129). Among antivenom-treated patients, the total antivenom dose was higher in the fasciotomy group (4.5 vials [IQR, 4–5] vs. 3 vials [IQR, 2–3], *p* < 0.001). Among patients who underwent fasciotomy, time to fasciotomy was 8 h [range, 7–8] after the bite, and the antivenom-to-fasciotomy interval was 2 h [range, 1–3]. Hospital length of stay was longer in patients who underwent fasciotomy (8 days [IQR, 6.75–9.25] vs. 1 day [IQR, 1–2], *p* < 0.001). In unadjusted exploratory comparisons of admission laboratory parameters, the fasciotomy group had a lower platelet count (174.5 [IQR, 174–175] vs. 219 [IQR, 176–270] × 10^3^/µL; *p* = 0.014), a longer aPTT (37.75 [IQR, 35.70–41.45] vs. 31.00 [IQR, 26.30–34.30] s; *p* = 0.027), and a lower serum creatinine level (0.61 [IQR, 0.58–0.74] vs. 0.86 [IQR, 0.70–0.96] mg/dL; *p* = 0.020). Hemoglobin, INR, and CK did not differ between the groups. Given the small number of fasciotomy cases and the multiple comparisons performed, these laboratory findings should be interpreted cautiously.

Exploratory correlations are presented in Table 4. The clinical profile score was positively correlated with bite-to-presentation time, bite-to-antivenom interval, and hospital length of stay. No statistically significant correlation was observed with age.

### 3.4. Profile and Complications Among Patients Undergoing Fasciotomy

All six patients who underwent fasciotomy had upper-extremity bites, comprising four forearm bites, one finger bite, and one hand bite, and all received antivenom before surgery. Patient-level clinical, operative, and postoperative characteristics are presented in Table 5. Fasciotomy wounds were closed with split-thickness skin grafts in all six patients. Mild superficial flexor muscle necrosis requiring superficial debridement was documented in one patient. One patient developed a surgical-site infection during the second postoperative week, which resolved with wound care and antibiotic treatment without additional surgery. No graft loss, persistent open wound, additional surgery after grafting, neurovascular deficit, or amputation was documented in the available postoperative follow-up records. However, follow-up timing and duration were not standardized, and range of motion, contracture, and functional recovery were not systematically assessed; therefore, long-term functional outcomes could not be evaluated.

## 4. Discussion

Within the complete-case subset, patients who underwent fasciotomy had more pronounced documented local findings during early clinical evaluation and presented later after the bite than those managed without fasciotomy. Among antivenom-treated patients in this subset, the fasciotomy group had a longer bite-to-antivenom interval and received a higher cumulative antivenom dose, whereas no statistically significant between-group difference was detected in the ED arrival-to-antivenom interval.

Snakebite envenomation can produce progressive swelling, pain, tenseness, paresthesia, and neurovascular abnormalities that may mimic acute compartment syndrome [4,9]. Previous studies have indicated that venom-induced compartment syndrome is uncommon and that substantial heterogeneity exists in diagnostic criteria, intracompartmental pressure measurement, and fasciotomy practices [5,6]. Consistent with these concerns, a recent global scoping review of 115 reported cases found that compartment pressure was measured in only 38% of patients and that reliance on clinical findings alone may contribute to overdiagnosis [10]. The review supported antivenom as first-line treatment but emphasized that evidence defining when fasciotomy should be used as rescue therapy remains limited [10].

The more pronounced local findings among patients who underwent fasciotomy are consistent with the prominence of pain and swelling in cases included in the global scoping review [10]. This similarity may reflect the local tissue effects of envenomation, which can overlap with findings used to suspect acute compartment syndrome [10,11,12]. However, pain with passive motion was documented in every patient in the complete-case subset and therefore did not distinguish the management groups. This observation is consistent with Pattinson et al., who emphasized that pain on passive stretch may occur after snakebite without necessarily indicating true compartment syndrome and should not be interpreted in isolation when assessing the need for fasciotomy [13]. The clinical profile score, which incorporated six documented local findings, should nevertheless remain interpreted as a descriptive summary rather than as a validated diagnostic tool or surgical threshold.

Within the complete-case subset, time from bite to emergency department presentation was positively correlated with the clinical profile score. This finding suggests that patients presenting later may have more pronounced local envenomation findings during early clinical evaluation. Local tissue effects after snakebite envenomation may evolve over time, and swelling, pain, tenseness, tissue injury, and neurovascular findings are important components of clinical monitoring [2]. Therefore, presentation timing should be considered not only as a measure of delay but also as a variable that should be interpreted together with the documented clinical severity of local findings during early evaluation. Nevertheless, because of the retrospective design and the use of emergency department records together with orthopedic consultation notes, this association should not be interpreted as causal or as reflecting findings present exclusively at emergency department arrival.

Antivenom timing and total dose may be influenced by multiple factors, including prehospital presentation delay, documented local findings during early clinical evaluation, progression during observation, and treatment requirements during follow-up [2]. Among the 21 antivenom-treated patients with reliable dose and timing data, the bite-to-antivenom interval was longer in the fasciotomy group, whereas the ED arrival-to-antivenom interval was not significantly different between groups. This distinction is important because the bite-to-antivenom interval reflects the overall time from snakebite to antivenom initiation and includes both the prehospital interval and in-hospital management time. Therefore, the longer bite-to-antivenom interval observed in the fasciotomy group appears to be largely related to later emergency department presentation rather than a clear delay in antivenom administration after hospital arrival. The higher cumulative antivenom dose observed in our fasciotomy group contrasts with Darracq et al., who found no statistically significant difference in total antivenom use between patients who underwent fasciotomy and those in whom fasciotomy was considered but not performed [14]. However, their study involved a selected cohort of rattlesnake-envenomated patients treated with a different antivenom product and dosing regimen; therefore, absolute vial counts are not directly comparable across studies, and the differing within-cohort patterns may reflect differences in case selection and treatment practices. Although recent preclinical testing demonstrated paraspecific neutralization by the HSGM polyvalent antivenom against *Vipera berus berus*, *Vipera ammodytes ammodytes*, and *Montivipera raddei* venoms [8], species-specific effectiveness and dose requirements could not be evaluated because snake species identification was unavailable. Among patients who underwent fasciotomy, surgery was performed 7–8 h after the bite and 1–3 h after antivenom initiation. The global scoping review reported a median antivenom-to-fasciotomy interval of 4 h (IQR, 0–13), although timing data were frequently missing [10]. Differences in case selection and clinical management limit direct comparison, and our retrospective records did not permit standardized assessment of the response to antivenom before surgery. Therefore, antivenom-related findings should be interpreted as descriptive treatment-timing and management characteristics rather than as causal determinants of fasciotomy.

The fasciotomy rate was 12.0% (6/50) in the overall extremity snakebite cohort and 17.1% (6/35) in the complete-case analytic cohort. Türkmen and Temel reported three fasciotomies among 97 patients with extremity snakebite (3.1%) managed using a structured classification and follow-up approach [15]. The higher proportion observed in our overall cohort may reflect differences in case mix and surgical decision-making. However, differences in assessment protocols and the absence of routine intracompartmental pressure measurement in our cohort limit direct comparison.

Hospital length of stay was also longer in patients who underwent fasciotomy, consistent with Darracq et al., who similarly reported longer hospitalization among patients undergoing fasciotomy [14]. This difference may reflect both greater clinical severity among patients selected for surgery and the additional wound and postoperative care associated with fasciotomy.

Admission laboratory comparisons were exploratory. The fasciotomy group had lower platelet counts, longer aPTT, and lower serum creatinine, whereas hemoglobin, INR, and CK did not differ significantly between groups. The global scoping review reported coagulopathy at admission in 40% of patients with reported venom-induced compartment syndrome [10], illustrating the coexistence of local and systemic manifestations of envenomation. However, that frequency cannot be directly compared with the between-group differences in platelet count and aPTT observed here, which do not by themselves establish coagulopathy. The clinical significance of the lower creatinine level remains uncertain. Given the small number of fasciotomy cases and multiple unadjusted comparisons, these laboratory findings should be interpreted cautiously and considered alongside clinical progression, bleeding risk, and response to antivenom rather than as isolated indications for surgery [2].

Given the diagnostic uncertainty, decisions regarding fasciotomy after extremity snakebite should incorporate a structured assessment rather than rely on isolated local examination findings. Serial neurovascular examinations, monitoring of local progression and clinical response to antivenom, and, when feasible, intracompartmental pressure measurement may help balance the risks of delayed decompression against unnecessary surgical intervention [10,16,17].

## 5. Limitations

This study has several important limitations. First, it was a single-center retrospective study; therefore, the findings depended on the accuracy and completeness of medical records, and external validity is limited. In addition, the overall cohort comprised only 50 patients, with six fasciotomy cases, and detailed clinical and treatment-timing analyses were restricted to a complete-case subset of 35 patients. This limited number of events precluded meaningful multivariable analysis and prevented independent or causal-effect interpretations regarding factors associated with fasciotomy performance. Restricting detailed analyses to the 35-patient complete-case subset may have introduced selection bias, particularly because all 15 patients omitted from these analyses were managed without fasciotomy. This may have affected the representativeness of the non-fasciotomy comparison group and the observed between-group differences; however, the direction and magnitude of any resulting bias could not be determined from the available data. Antivenom administration status was unknown in 11 patients, which also limits interpretation of the available-case comparison of antivenom administration.

Second, because intracompartmental pressure measurements were not routinely performed or documented, objective confirmation of acute compartment syndrome was not possible, and fasciotomy was therefore analyzed as an observed management outcome rather than as confirmation of the diagnosis. In addition, the snake species could not be identified in any patient in the complete-case subset; given the documented variation in venom composition among Turkish viper taxa [18], the findings could not be attributed to a specific snake species, venom profile, or species-specific clinical course.

Laboratory analyses were based on initial or same-day admission values and did not capture serial changes in coagulation parameters, muscle injury markers, or renal function. Fibrinogen and D-dimer were available for only 7 and 12 patients, respectively, while separate PT and fibrin degradation product measurements were not consistently available; therefore, these parameters could not be included in between-group comparisons. The small number of fasciotomy cases and the use of multiple unadjusted comparisons further limit the robustness of the observed laboratory differences.

In addition, the retrospective records did not permit standardized assessment of clinical progression or response to antivenom before fasciotomy. Intraoperative muscle viability was incompletely documented; mild superficial flexor muscle necrosis was recorded in one patient, whereas detailed muscle viability findings were unavailable for the remaining five patients. The records also did not provide a standardized account of the precise rationale underlying each surgical decision or the timing of split-thickness skin grafting. Furthermore, the timing and duration of postoperative follow-up were not standardized, and range of motion, contracture, and functional recovery were not systematically assessed; therefore, long-term functional outcomes could not be evaluated.

Finally, the clinical profile score was not a validated snakebite-specific score; it was retrospectively constructed to standardize bedside clinical findings documented during early clinical evaluation. Because these findings were extracted from emergency department records and orthopedic consultation notes, the score may reflect both initial clinical status and findings documented during subsequent evaluation before the final management decision. Furthermore, the association between the clinical profile score and fasciotomy may partly reflect the influence of its component findings on surgical decision-making. Accordingly, our results should be considered hypothesis-generating and require confirmation in prospective, multicenter studies incorporating serial clinical assessment and objective pressure measurement when feasible.

## 6. Conclusions

In the complete-case subset of this single-center retrospective cohort, patients who underwent fasciotomy had more pronounced documented local findings and later presentation than those managed without fasciotomy. Among antivenom-treated patients in this subset, cumulative doses were higher, and the bite-to-antivenom interval was longer in the fasciotomy group, whereas no statistically significant difference was detected in the ED arrival-to-antivenom interval. These descriptive findings characterize patients selected for fasciotomy but do not establish pressure-confirmed acute compartment syndrome or the necessity of surgery. Prospective studies incorporating standardized serial clinical assessments, documentation of the response to antivenom, and objective compartment pressure measurement when feasible are needed to better evaluate surgical decision-making.

## Figures and Tables

**Table 1:** Characteristics of the overall extremity snakebite cohort and complete-case subset.

Variable	Overall Cohort (n = 50)	Complete-Case Subset (n = 35)
Age, years, median (IQR)	47 (29.5–60)	50 (27–60)
Sex, n (%)		
Female	20 (40.0)	16 (45.7)
Male	30 (60.0)	19 (54.3)
Bitten extremity, n (%)		
Upper extremity	37 (74.0)	22 (62.9)
Lower extremity	13 (26.0)	13 (37.1)
Antivenom administration status, n (%)		
Administered	25 (50.0)	21 (60.0)
Not administered	14 (28.0)	14 (40.0)
Unknown	11 (22.0)	0 (0.0)
Fasciotomy performed, n (%)	6 (12.0)	6 (17.1)
Clinical profile score [0–6], median (IQR)	-	1 (1–3)
Time from bite to ED presentation, h, median (IQR)	-	2 (2–3)
Length of hospital stay, days, median (IQR)	-	1 (1–3)

Values are presented as number (%) or median (interquartile range). The complete-case subset comprises 35 of the 50 patients in the overall cohort. Percentages use the corresponding cohort size as the denominator. A dash indicates that an overall-cohort summary was unavailable. Unknown antivenom administration status indicates insufficient documentation and was not classified as non-administration. Abbreviations: ED, emergency department; IQR, interquartile range.

**Table 2:** Individual documented local clinical findings during early clinical evaluation according to fasciotomy management.

Variable	Fasciotomy Performed (n = 6)	No Fasciotomy (n = 29)	*p* Value
Pain at rest, n (%)	6 (100.0)	3 (10.3)	<0.001
Pain with passive motion, n (%)	6 (100.0)	29 (100.0)	1.000
Marked swelling or tenseness, n (%)	6 (100.0)	3 (10.3)	<0.001
Paresthesia, n (%)	6 (100.0)	2 (6.9)	<0.001
Motor deficit, n (%)	6 (100.0)	0 (0.0)	<0.001
Delayed capillary refill, n (%)	5 (83.3)	0 (0.0)	<0.001
Clinical profile score during early clinical evaluation [0–6], median (IQR)	6 (5.75–6.00)	1 (1–1)	<0.001

Values are presented as number (%) or median (interquartile range). Percentages are column percentages. Fisher’s exact test was used for categorical variables. Each finding explicitly documented as present was assigned 1 point, and each finding explicitly documented as absent was assigned 0 points, yielding a total clinical profile score of 0–6. Missing documentation was not coded as absence. Item definitions are provided in Section 2. The score was used solely as a descriptive summary, not as a diagnostic tool or surgical decision threshold.

**Table 3:** Demographic, treatment-timing, laboratory, and hospital-course characteristics according to fasciotomy management.

Variable	Fasciotomy Performed	No Fasciotomy	*p* Value
Overall cohort, n	6	44	
Age, years, median (IQR)	30.5 (23–58.5)	48.5 (33.25–60.75)	0.244
Sex, n (%)			0.672
Female	3 (50.0)	17 (38.6)	
Male	3 (50.0)	27 (61.4)	
Bitten extremity, n (%)			0.319
Upper extremity	6 (100.0)	31 (70.5)	
Lower extremity	0 (0.0)	13 (29.5)	
Patients with known antivenom administration status, n	6	33	
Antivenom administration, n (%)			0.071
Administered	6 (100.0)	19 (57.6)	
Not administered	0 (0.0)	14 (42.4)	
Complete-case subset, n	6	29	
Time from bite to ED presentation, hours, median (IQR)	4.5 (4–5)	2 (2–2)	<0.001
Hospital length of stay, days, median (IQR)	8 (6.75–9.25)	1 (1–2)	<0.001
Admission platelet count, ×10^3^/µL, median (IQR)	174.5 (174–175)	219 (176–270)	0.014
Admission hemoglobin, g/dL, median (IQR)	12.25 (10.90–16.53)	14.00 (12.80–16.00)	0.584
Admission INR, median (IQR)	0.97 (0.96–1.51)	0.95 (0.93–1.05)	0.443
Admission aPTT, seconds, median (IQR)	37.75 (35.70–41.45)	31.00 (26.30–34.30)	0.027
Admission CK, U/L, median (IQR)	261.5 (181–317.3)	161 (91.2–356)	0.444
Admission creatinine, mg/dL, median (IQR)	0.61 (0.58–0.74)	0.86 (0.70–0.96)	0.020
Antivenom-treated patients in the complete-case subset, n	6	15	
Bite-to-antivenom interval, hours, median (IQR)	4.5 (4–5)	2 (2–3)	<0.001
ED arrival-to-antivenom interval, hours, median (IQR)	0 (0–0)	0 (0–1)	0.129
Total antivenom dose, vials, median (IQR)	4.5 (4–5)	3 (2–3)	<0.001
Fasciotomy-specific timing			
Time from bite to fasciotomy, hours, median (range)	8 (7–8)	Not applicable	-
Antivenom-to-fasciotomy interval, hours, median (range)	2 (1–3)	Not applicable	-

Values are presented as number (%), median (interquartile range), or median (range), as indicated. Sample sizes are shown for each analysis block; percentages use the corresponding group denominator. Age, sex, and bitten extremity were analyzed in the overall cohort (n = 50). Antivenom administration was compared among patients with known status (n = 39); 11 patients without fasciotomy had unknown status and were excluded from this comparison. Clinical, laboratory, and hospital-course comparisons used the complete-case subset (n = 35). Antivenom dose and timing analyses included only treated patients within this subset (n = 21); four additional patients had documented administration but lacked reliable dose and timing data. The Mann–Whitney U test was used for continuous variables and two-sided Fisher’s exact tests for categorical variables. Fasciotomy-specific intervals were summarized descriptively without between-group testing. All *p*-values are unadjusted and exploratory. Abbreviations: aPTT, activated partial thromboplastin time; CK, creatine kinase; ED, emergency department; INR, international normalized ratio; IQR, interquartile range.

**Table 4:** Exploratory correlations between the clinical profile score and selected demographic, timing, and hospital-course variables.

Variable Correlated with the Clinical Profile Score	Spearman’s ρ	*p*-Value	n
Time from bite to emergency department presentation	0.607	<0.001	35
Bite-to-antivenom interval	0.794	<0.001	21
Hospital length of stay	0.752	<0.001	35
Age	−0.296	0.084	35

Spearman’s rank correlation was used. The bite-to-antivenom analysis included only antivenom-treated patients (n = 21); all other analyses included the complete-case cohort (n = 35). Analyses were exploratory, and *p*-values were not adjusted for multiple comparisons. ρ, Spearman’s rank correlation coefficient; n, number of patients included in each analysis.

**Table 5:** Patient-level clinical, treatment, operative, and outcome characteristics of patients who underwent fasciotomy.

Patient	Bite Site	Documented Local Findings Before the Management Decision	Antivenom Timing/Total Dose	Documented Reason for Fasciotomy	Documented Extent of Fasciotomy	Intraoperative Findings, Tissue Necrosis, and Debridement	Wound Management, Available Postoperative Follow-Up, and Outcome
1	Forearm	All six predefined findings	5 h/5 vials	Clinical concern for compartment syndrome	Forearm and hand; individual compartments not specified	Mild necrosis of the superficial flexor musculature of the forearm; superficial debridement performed; no further tissue necrosis documented	Wound closed with a split-thickness skin graft; no graft loss, persistent open wound, or additional surgery after grafting; no neurovascular deficit documented during available postoperative follow-up; no amputation
2	Forearm	All six predefined findings	4 h/4 vials	Clinical concern for compartment syndrome	Forearm and hand; individual compartments not specified	No tissue necrosis or need for debridement documented; detailed muscle viability findings unavailable	Wound closed with a split-thickness skin graft; no graft loss, persistent open wound, or additional surgery after grafting; no neurovascular deficit documented during available postoperative follow-up; no amputation
3	Forearm	All six predefined findings	4 h/5 vials	Clinical concern for compartment syndrome	Forearm and hand; individual compartments not specified	No tissue necrosis or need for debridement documented; detailed muscle viability findings unavailable	Wound closed with a split-thickness skin graft; surgical-site infection developed during postoperative week 2 and resolved with wound care and antibiotic treatment without additional surgery; no graft loss or persistent open wound; no neurovascular deficit documented during available postoperative follow-up; no amputation
4	Forearm	All six predefined findings	5 h/4 vials	Clinical concern for compartment syndrome	Forearm and hand; individual compartments not specified	No tissue necrosis or need for debridement documented; detailed muscle viability findings unavailable	Wound closed with a split-thickness skin graft; no graft loss, persistent open wound, or additional surgery after grafting; no neurovascular deficit documented during available postoperative follow-up; no amputation
5	Finger	All six predefined findings	4 h/5 vials	Clinical concern for compartment syndrome	Forearm and hand with additional decompression of the second finger; individual compartments not specified	No tissue necrosis or need for debridement documented; detailed muscle viability findings unavailable	Wound closed with a split-thickness skin graft; no graft loss, persistent open wound, or additional surgery after grafting; no neurovascular deficit documented during available postoperative follow-up; no amputation
6	Hand	Five predefined findings; delayed capillary refill documented as absent	5 h/4 vials	Clinical concern for compartment syndrome	Forearm and hand; individual compartments not specified	No tissue necrosis or need for debridement documented; detailed muscle viability findings unavailable	Wound closed with a split-thickness skin graft; no graft loss, persistent open wound, or additional surgery after grafting; no neurovascular deficit documented during available postoperative follow-up; no amputation

Note: The predefined local findings were pain at rest, pain with passive motion, marked swelling or tenseness, paresthesia, motor deficit, and delayed capillary refill. Findings were extracted from a single documentation series comprising emergency department and/or orthopedic assessment before the final management decision; standardized serial progression data were unavailable. Intracompartmental pressure measurement was not performed or documented in any patient, and snake species could not be identified. Fasciotomy wounds were closed with split-thickness skin grafts in all six patients, although the timing of grafting was not reliably documented. The timing and duration of postoperative follow-up were not standardized and could not be reliably determined on a patient-by-patient basis. Range of motion, contracture, and functional recovery were not systematically assessed or documented; therefore, long-term functional outcomes could not be evaluated.

## Data Availability

The datasets generated and/or analyzed during the current study are not publicly available because they contain de-identified clinical data subject to institutional and ethical restrictions but are available from the corresponding author upon reasonable request and with appropriate institutional permission.

## Abbreviations

aPTT	Activated partial thromboplastin time
CK	Creatine kinase
ED	Emergency department
HSGM	Halk Sağlığı Genel Müdürlüğü (General Directorate of Public Health, Türkiye)
IBM	International Business Machines
ICD-10	International Classification of Diseases, 10th Revision
INR	International normalized ratio
IQR	Interquartile range
PT	Prothrombin time
SPSS	Statistical Package for the Social Sciences
US/USA	United States/United States of America
